# Influence of AgVO_3_ incorporation on antimicrobial properties, hardness, roughness and adhesion of a soft denture liner

**DOI:** 10.1038/s41598-019-48228-8

**Published:** 2019-08-15

**Authors:** Simone Kreve, Viviane C. Oliveira, Luciano Bachmann, Oswaldo L. Alves, Andréa C. Dos Reis

**Affiliations:** 1 Department of Dental Materials and Prosthodontics, Ribeirão Preto Dental School, USP—University of São Paulo, Ribeirão Preto, SP Brazil; 2 Technique of Oral Rehabilitation Laboratory, Ribeirão Preto Dental School, University of São Paulo, Ribeirão Preto, Brazil; 3 Department of Physics, University of São Paulo - School of Philosophy, Sciences and Letters of Ribeirão Preto, Ribeirão Preto, São Paulo, Brazil; 4 Laboratory of Solid State Chemistry, State University of Campinas (UNICAMP), Campinas, Brazil

**Keywords:** Quality of life, Dental biomaterials

## Abstract

The objective of this *in vitro* study was to investigate the effect of nanostructured silver vanadate decorated with silver nanoparticles (AgVO_3_) on antimicrobial activity, hardness, roughness, and adhesion of a soft denture liner. The antimicrobial efficacy of the Trusoft (Boswoth) liner incorporated with different concentrations of AgVO_3_ against *Enterococcus faecalis*, *Pseudomonas aeruginosa*, *Candida albicans*, and *Staphyloccocus aureus* (n = 5) was evaluated by the agar diffusion method. Roughness, hardness, and adhesion properties were also evaluated. The data were analyzed by analysis of variance (ANOVA) and Tukey’s multiple comparison test with significance at the p < 0.05 level. At concentrations of 1 and 2.5%, AgVO_3_ incorporation was effective only against *E*. *faecalis*, and at 5 and 10%, against *E*. *faecalis*, *P*. *aeruginosa*, and *C*. *albicans*. None of the concentrations was effective against *S*. *aureus*. A decrease in hardness was found for the 1, 2.5, and 10% AgVO_3_ concentrations (p < 0.001) and at 5%, hardness was not affected. None of the concentrations affected the roughness of the material. A significant increase in tensile values was observed between the liner and heat-curing acrylic resin for 2.5% (p < 0.001) and 10% (p = 0.042) concentrations. AgVO_3_ incorporation to a soft denture liner promoted antimicrobial activity against *E*. *faecalis*, *P*. *aeruginosa*, and *C*. *albicans* without affecting roughness, maintaining the hardness properties recommended for soft and extra soft liners, and improving the adhesion between the liner and the acrylic resin used for dentures.

## Introduction

Soft denture liners based on poly-methyl methacrylate or silicone elastomers are used for repairs, rebuilding the surface of the prosthesis in contact with the oral mucosa^1–3^, and reducing forces to supporting tissues^4–7^. These materials are recommended for patients with extremely resorbed residual ridges^3,4,8^, and for implant osseointegration^1,9^ as they promote an even distribution of the supporting loads. However, because of their structural form, composition, and properties, soft liners have a porous surface^1,10^ and thus function as microbial reservoirs^10^. Liner contamination may cause serious local problems such as implant loss^1,11^, peri-implant infections^11,12^, delay in implant osseointegration, pain^13^, and discomfort, in addition to systemic diseases such as bacterial endocarditis, pneumonia, chronic obstructive pulmonary disease, and oropharyngeal, esophageal, and respiratory infections^14–17^ interfering with treatment success, general health, and quality of life. Thus, the addition of antimicrobial properties to denture liners would have many advantages.

The antimicrobial property of denture liners is usually achieved with the incorporation of antimicrobial agents, which often causes changes in the physical-chemical and mechanical properties of the materials, hindering their use. The incorporation of ketoconazole, chlorhexidine, nystatin, and miconazole have shown to cause significant increase in the material’s hardness (although itraconazole did not affect hardness)^18^. The use of silver nanoparticles caused a decrease in adhesion to the prosthesis, modified the type of adhesive failure, and decreased the hardness of the material with increasing concentrations of antimicrobial agent^19^. Inhibition of *C*. *albicans* and reduction of surface roughness was observed with the incorporation of essential oils^20^. The addition of silver nanoparticles to a tissue conditioner in concentrations varying between 0.1 and 3.0% was effective against *S*. *aureus*, *S*. *mutans* (at 0.1%), and *C*. *albicans* (at 0.5%) after a period of 24 and 72 h^16^.

To add antimicrobial properties to denture liners while maintaining good physical and chemical properties, this study tested the incorporation of nanostructured silver vanadate, since its use was shown to be efficient in acrylic resin^21,22^.

Nanostructured silver vanadate decorated with silver nanoparticles (AgVO_3_) is formed by a precipitation reaction between silver nitrate and ammonia vanadate and was described by Holtz *et al*.^23,24^. This substance showed promising antibacterial activity against strains of gram-positive bacteria, including methicillin-resistant *S*. *aureus*.

Therefore, the objective of this study was to incorporate AgVO_3_ at 1, 2.5, 5, and 10% to a resin denture liner and evaluate the antimicrobial capacity, adhesion properties, hardness, and roughness. The null hypothesis was that there is no significant difference in the properties of the liner and antimicrobial effectiveness as a function of AgVO_3_ concentration.

## Material and Method

### Synthesis of nanostructured silver vanadate

The nanostructured silver vanadate is synthesized through a precipitation reaction between silver nitrate (AgNO_3_, Merck 99.8%) and ammonium vanadate (NH_4_VO_3_, Merck 99%), as described previously^21–24^. After the solubilization of 0.9736 g of NH_4_VO_3_ and 1.3569 g of AgNO_3_ in 200 mL of distilled water, the AgNO_3_ solution was added dropwise to the NH_4_VO_3_ solution under constant stirring at 65 °C. The obtained precipitate was washed with distilled water and absolute alcohol, filtered, and dried under vacuum for 10 hours^23,24^.

### Materials characterization

#### Analysis of transmission electron microscopy (TEM)

**Figure 1:**
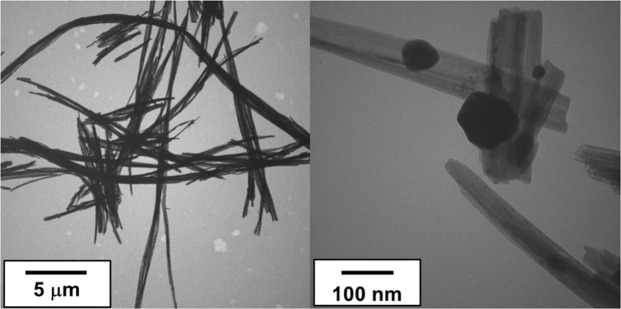
TEM images of AgVO_3_ decorated with AgNPs (5000 and 200000X magnification).

The presence and morphology of silver nanoparticles (AgNPs) on the surface of the crystals formed was verified by transmission electron microscopy (TEM) using the JEOL microscope JEM-100CX II (Fig. 1).

#### Analysis of Fourier Transform Infrared (FTIR) spectroscopy

**Figure 2:**
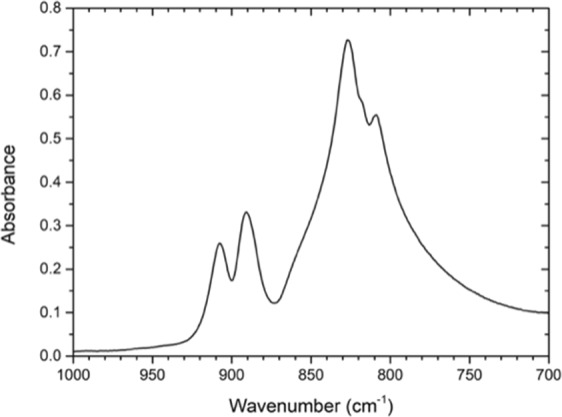
FTIR spectra in the wavenumber range 400–4000 cm^−1^ of the as synthesized AgVO_3_.

The chemical composition of AgVO_3_ was evaluated by Fourier Transform Infrared (FTIR) spectroscopy; that was performed using ATR technique with a diamond crystal detector coupled to the FTIR Spectrometer Nicolet 380 (Thermo Fisher Scientific Inc.; Waltham, MA, USA). For the measurement, the AgVO_3_ were positioned in contact with the diamond crystal of the ATR setup. The infrared absorbance spectrum of the samples was obtained over the range of 4000–400 cm^−1^ at 1 cm^−1^ resolution using 32 scans and was recorded using the OMNIC Spectra Software (Fig. 2).

For the peak identification we employ the second derivative to the absorption spectrum to identify precisely the band position.

#### Analysis of X-ray diffraction (XRD)

**Figure 3:**
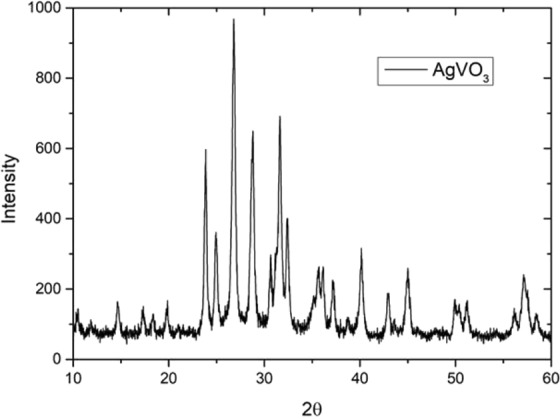
X-ray diffraction pattern of silver vanadate nanostructure.

The structural changes were evaluated by x-ray diffraction (XRD) patterns. The patterns were collected using a Bruker AXS D2 diffractometer equipped with a Cu tube, using Cu Kα radiation (λ = 1.54 Å); in the range of 5–50°, with steps of 0.05°, with 0,5 s for each step (Fig. 3).

### Preparation of the resin liner and specimens containing AgVO_3_

The powder of Trusoft (Boswoth) denture liner was weighed to obtain the amount of nanomaterial to be added. Fractions from 1 to 10% of the liner weight were subtracted to incorporate that amount of the nanomaterial, as described in Table 1.

**Table 1:** Composition of tested resin liners.

**Composition (%mass)**
Resin liner
Resin liner + 1% AgVO_3_
Resin liner + 2.5% de AgVO_3_
Resin liner + 5% de AgVO_3_
Resin liner + 10% de AgVO_3_

For the antimicrobial, hardness and roughness analyses, the AgVO_3_ + liner combinations were mixed for 30 to 40 seconds on an unpolished glass slab. The mix was placed in a metal mold (Ø 8 mm × 3 mm) and pressed between two glass plates to create sample disks. The entire procedure was done according to the manufacturer’s recommendations.

For the adhesion analysis, a mold with 10 mm diameter and 20 mm height was used to obtain specimens in wax that were invested with plaster in metal flasks, creating a matrix for packing with heat or self-curing acrylic resin (Clássico Produtos Odontológicos, SP, Brazil). One hundred disks of each acrylic resin were obtained, which were polished under water with 320-grit sandpaper. Two disks of each resin type were positioned in a spacer and joined with the liner, generating 50 specimens for each resin (Ø10 mm × 43 mm, n = 10 for each percentage of nanomaterial).

### Analysis of antimicrobial activity

The microorganisms *Staphylococcus aureus* (ATCC 6538), *Pseudomonas aeruginosa* (ATCC 27853), *Candida albicans* (ATCC 90028), and *Enterococcus faecalis* (ATCC 29212) were obtained from thawed strains and cultured at 37 °C for 24 hours in a microbiological oven. Cultures were centrifuged at 6,000 *g* for 5 minutes, the supernatant was discarded, and the pellet was washed twice with 10 mL of PBS (Phosphate Buffered Saline, containing NaCl, KCl, Na_2_HPO_4_, KH_2_PO_4_). The inoculum suspensions were made in BHI medium and the concentration confirmed by optical density using a spectrophotometer (BEL Photonics, model 1105, Piracicaba, SP, Brazil), with absorbance reading of 0.120 for *P*. *aeruginosa*, 0.150 for *E*. *faecalis*, 0.8 for *S*. *aureus* at 625 nm wavelength, which corresponds to 10^8^ cells/mL. For *C*. *albicans*, cells were counted in the Newbauer chamber due to the variable morphology of the genus. After standardization of the suspensions, the inocula were diluted to 10^6^ CFU/mL.

TSA (tryptic soy agar) culture medium was used for *P*. *aeruginosa* and *E*. *faecalis*, Saboraud Dextrose Agar was used for *C*. *albicans*, and BHI (Brain Heart Infusion) was used for *S*. *aureus*.

To evaluate the 4 microorganisms, the agar diffusion method was performed with 5 specimens for each percentage of nanomaterial. Denture liner disks (Ø 8 mm × 3 mm) were exposed to UV light in a laminar flow hood for 30 min on each side for disinfection and then placed in petri dishes containing the culture media. The culture media for diffusion were heated to 50 °C for addition of the microorganisms. After 2-h rest, dishes were stored for 24 hours in an oven at 37 °C.

### Hardness and roughness tests

The surface hardness analysis was performed using the microdurometer Microtest SD 300 (Moema, SP, Brazil) with five random equidistant measurements in each specimen using a Shore A indenter. The roughness was analyzed using the Mitutoyo analogue roughness tester (Mitutoyo Suzano, São Paulo, Brazil) with three measurements in each specimen: one in the center and two 1-mm from the central measurement.

### Tensile test

The tensile test was performed according to ISO 20795-1: 2008 in a universal testing machine (EMIC, São José dos Pinhais, Paraná, Brazil) with a speed of 5 mm/min. Data were collected using Tesc^®^ software v.3.01 (EMIC).

The failure types of the test groups were observed and classified as adhesive failure - total separation at the interface between the liner and the acrylic resin; cohesive failure - separation within the liner; and mixed failure – a mix of adhesive and cohesive failures.

Tensile bond strength was calculated using the equations below:

$${\rm{\sigma }}=F/A$$

where σ = stress (MPa), F = maximum recorded strength at failure (N), and A = original cross sectional area (mm^2^).

### Data analysis

After verifying the normal distribution of the data (Kolmogorov-Smirnov test), ANOVA with Tukey’s Multiple Comparison test (α = 0.05) was applied for comparisons and the Two Proportions Equality test to analyze the distribution of the relative frequencies (percentages). The software used was SPSS v 20.0.

## Results

### FTIR, SEM and X-ray diffraction (XRD) analysis

The AgVO_3_ morphology was examined by TEM, and it shows that the synthesized sample is mostly rod-like particles (Fig. 1). The AgVO_3_ are composed of nanowires with length in the order of micrometers. The wires are coated with semispherical metallic silver particles (Ag).

**Figure 4:**
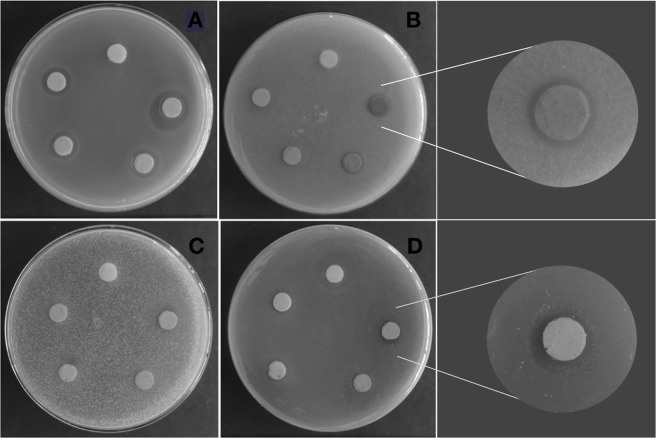
Antimicrobial activity of AgVO_3_ in different concentrations against different microorganisms. *A, *E*. *faecalis*; B, *C*. *albicans*; C, *S*. *aureus*; D *P*. *aeruginosa*. The specimens at the top of each dish are the control (0% AgVO_3_), and counter-clockwise are the 1, 2.5, 5, and 10% AgVO_3_.

Figure 4 shows the fourier transform infrared (FTIR) spectrum of AgVO_3_ nanorods acquired in the range of 400–4000 cm^−1^ which is very similar to that reported for AgVO_3_^22,23^.

It was observed absorption bands at 908 cm^−1^, 892 cm^−1^, 860 cm^−1^ (broad band), 827 cm^−1^, 817 cm^−1^ and 809 cm^−1^. We didn’t find the same bands in the literature, but for the same Ag/V molar ratio of the two used in this work, it was observed bands at 964, 915, 895, and 849 cm^−1^ ^23^. These absorption bands area was assigned to stretching vibration mode of the V = O bound.

In Fig. 3 it is observed the x-ray diffraction pattern of the silver vanadate. The x-ray diffraction pattern is a mean value of silver vanadate nanowires who are decorated with Ag nanoparticles. The data depicts that the sample is crystalline, but we didn’t identify in this work its crystalline structure, remembering that we have certainly at least two-phase as suggested by the literature^23^, metallic Silver (Ag°) and silver vanadate (AgVO_3_).

### Antimicrobial activity

Antimicrobial activity was tested by the Kirby Bauer agar diffusion method. The incorporation of 1 and 2.5% AgVO_3_ to the denture liner did not result in antimicrobial activity for *P*. *aeruginosa* and *C*. *albicans*, but it was effective at 5 and 10%; for *P*. *aeruginosa*, the 10% showed higher antimicrobial capacity. For *C*. *albicans*, both 5 and 10% concentrations showed similar antimicrobial effect. All preparations showed antimicrobial activity for *E*. *faecalis* in a concentration-dependent manner (with 10% having the highest effect). None of the concentrations was effective against *S*. *aureus* (Fig. 4).

### Mechanical properties

**Figure 5:**
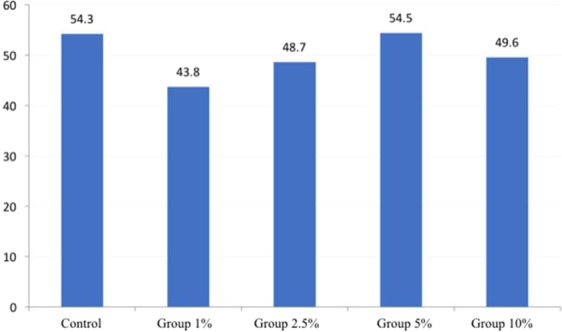
Hardness Shore A values for the control group and experimental groups with different concentrations of AgVO_3_.

The incorporation of 1, 2.5, and 10% AgVO_3_ promoted a decrease in Shore A values (p < 0.001), while the 5% dose had no effect. The incorporation of 1% AgVO_3_ produced specimens with a lower Shore A hardness value (average of 43.77), which is within the ISO standard of 40 units (DIN 53505 and ASTM D2240 / 75) for soft denture liners (Fig. 5).

The addition of the nanomaterial at all concentrations did not significantly affect the roughness of the denture liner. (Table 2).

**Table 2:** Mean, median, and standard deviation for the roughness property of a denture liner incorporated with nanostructured silver vanadate at different concentrations.

Roughness	Mean	Median	SD	CV	Min	Max	N	IC	P-value
Control	5.99	6.16	0.83	14%	4.18	7.48	20	0.36	0.144
Group 1%	6.24	6.21	0.82	13%	4.38	8.26	20	0.36	
Group 2.5%	6.56	6.55	0.85	13%	5.31	8.19	20	0.37	
Group 5%	6.17	6.03	0.71	11%	4.96	7.85	20	0.31	
Group 10%	5.84	6.07	1.28	22%	2.12	7.94	20	0.56	

For the heat-curing acrylic resin, a significant increase in tensile values was found for 2.5% (p < 0.001) and 10% AgVO_3_ (p = 0.042), showing that the nanomaterial caused an increase in the adhesive property of the liner (Table 3). The 2.5% concentration promoted the highest adhesion with a mean of 0.664 MPa. For the self-curing acrylic resin, no significant effect was found in strenght (N/cm and Kgf) and tension (MPa) in any of the groups evaluated (Table 4).

**Table 3:** Mean, median, and standard deviation (SD) for tensile values between a denture liner incorporated with nanostructured silver vanadate at different concentrations and a heat-curing acrylic resin.

		Mean	Median	SD	CV	Min	Max	N	95%CI	P-value
Max. Strength Máx (N)	Control	39.5	38.6	5.8	15%	30.6	49.8	10	3.6	<0.001
	Group 1%	45.5	44.8	5.7	12%	36.9	52.5	10	3.5	
	Group 2.5%	52.1	52.8	6.8	13%	39.6	60.5	10	4.2	
	Group 5%	41.9	41.8	5.3	13%	34.9	51.0	10	3.3	
	Group 10%	47.0	47.4	5.0	11%	36.9	53.9	10	3.1	
Max. Strength (Kgf)	Control	4.03	3.94	0.59	15%	3.12	5.08	10	0.36	<0.001
	Group 1%	4.64	4.57	0.58	12%	3.76	5.35	10	0.36	
	Group 2.5%	5.31	5.38	0.69	13%	4.04	6.17	10	0.43	
	Group 5%	4.28	4.26	0.54	13%	3.56	5.20	10	0.34	
	Group 10%	4.79	4.83	0.51	11%	3.76	5.50	10	0.32	
Tensile strength (MPa)	Control	0.504	0.50	0.073	15%	0.39	0.63	10	0.045	<0.001
	Group 1%	0.580	0.57	0.072	12%	0.47	0.67	10	0.045	
	Group 2.5%	0.664	0.68	0.086	13%	0.50	0.77	10	0.053	
	Group 5%	0.533	0.53	0.069	13%	0.44	0.65	10	0.043	
	Group 10%	0.599	0.61	0.063	11%	0.47	0.69	10	0.039

**Table 4:** Mean, median and standard deviation (SD) for tensile values between a denture liner incorporated with nanostructured silver vanadate at different concentrations and a self-curing acrylic resin.

Tração Auto		Mean	Median	SD	CV	Min	Max	N	IC	P-valor
Strength Max (N)	Control	42.7	44.0	9.8	23%	26.8	59.3	10	6.1	0.020
	Group 1%	46.0	40.4	16.8	37%	25.7	68.3	9	11.0	
	Group 2,5%	34.2	30.6	21.7	63%	11.2	68.4	10	13.4	
	Group 5%	23.9	21.6	10.6	44%	11.2	41.0	10	6.6	
	Group 10%	29.8	21.7	16.0	54%	13.0	56.2	9	10.4	
Strength Max (Kgf)	Control	4.36	4.49	1.00	23%	2.73	6.05	10	0.62	0.020
	Group 1%	4.70	4.12	1.72	37%	2.62	6.96	9	1.12	
	Group 2,5%	3.49	3.13	2.21	63%	1.14	6.98	10	1.37	
	Group 5%	2.44	2.20	1.08	44%	1.15	4.18	10	0.67	
	Group 10%	3.04	2.21	1.63	54%	1.32	5.73	9	1.06	
Strength (MPa)	Control	0.544	0.56	0.127	23%	0.34	0.76	10	0.079	0.021
	Group 1%	0.584	0.51	0.214	37%	0.33	0.87	9	0.140	
	Group 2,5%	0.436	0.39	0.276	63%	0.14	0.87	10	0.171	
	Group 5%	0.304	0.28	0.135	44%	0.14	0.52	10	0.084	
	Group 10%	0.382	0.28	0.203	53%	0.17	0.72	9	0.133	

Regarding the type of failure for the heat-curing resin, a significant difference was found in the 2.5% group, with 50% of mixed failures and the 10% group with 60% of adhesive failures. For the self-curing resin, a difference was found only in the 10% group, with 80% of adhesive failures (p = 0.007) (Table 5). When comparing the two resin types, a significant difference was found only for the 2.5% group (p = 0.010), with 50% of failures within the heat-curing resin.

**Table 5:** Comparison of failure type between heat- and self-curing acrylic resin and a denture liner incorporated with nanostructured silver vanadate in different concentrations.

		Control		Group 1%		Group 2,5%		Group 5%		Group 10%	
		N	%	N	%	N	%	N	%	N	%
Self-curing resin	Adhesive	2	20%	0	0%	0	0%	2	20%	8	80%
	Cohesive	8	80%	10	100%	10	100%	8	80%	2	20%
Heat-curing resin	Adhesive	0	0%	0	0%	5	50%	1	10%	6	60%
	Cohesive	10	100%	10	100%	5	50%	9	90%	4	40%

## Discussion

The AgVO_3_ formed had acicular morphology and length in the order of micrometers, and they are decorated with AgNPs, similar to the pattern observed in the images obtained by transmission electron microscopy (TEM) by de Castro *et al*.^22^ and Holtz *et al*.^24^. The characterization showed a vanadate wire with a heterogeneous distribution and few AgNPs decorating the surface of the nanowires, and both vanadate and Ag present antibacterial effect, which characterizes the antibacterial potential of the material.

The infrared absorption bands show very broad bands, and different works from the literature diverge from the nominal values of the bands position observed in this work. These differences can be assigned for different Ag/V molar ratio and also to the synthesis.

AgVO_3_ is a promising alternative to provide antimicrobial capacity to various materials^23^ such as water-based paints^24^ and, in dentistry, acrylic resin^21,22^.

In relation to the efficacy of silver vanadate antimicrobial properties it was seen that the antimicrobial mechanism of AgVO3 is attributed to the contact between the hybrid nanomaterial and bacteria^22^ and occurs when Ag + is absorbed and released from the Ag nanoparticles through a direct dissociation between the nanoparticle and the bacterial cell wall, causing toxic effects^23,24^. Besides that, the AgVO_3_ maintains a large surface area in contact with microorganisms, promoting changes in bacterial membranes morphology and loss of DNA’s ability to replicate, which can cause cell death^23^. The surface of the bacterium has a negative charge at biological pH due to the dissociation of a large number of functional groups, including carboxylic and other chemical groups on the membrane. According to Li *et al*.^25^ the semiconductor antibacterial mechanism is attributed to the oxidative stress caused by the production of OH*, O_2_ and and O_2_H *, that is, when reactive species with complex groupings such as AgO_3_VO are formed a cluster in the reaction where O_3_ decomposes into hydroxyl radicals and protons. At the same time, an electron transferred to the molecule of O interacts with the proton, forming an O_2_H* radical. Thus, bacterial death occurs through collaboration between surfaces^26^.

In this study, nanostructured silver vanadate was incorporated into a resin denture liner and the following properties were evaluated: antimicrobial effectiveness, roughness, hardness, and adhesion. The null hypothesis that the addition of AgVO_3_ does not significantly affect the properties of the liner and antimicrobial effectiveness in a concentration-dependent manner was partially accepted.

Soft resins used for denture lining can serve as reservoirs for microorganisms^1^ even when hygienized^10^ due to their porous and irregular surface. Thus, research has been carried out for finding antimicrobial possibilities^1,6,20,26–28^ to simultaneously treat infected mucosa and decrease denture fungal infections^29^. However, antimicrobial agents generally affect the physicochemical and mechanical properties of the material^6,18^. Denture liners contamination favors numerous diseases of the respiratory tract that cause serious health problems^14,17^. Even with the proven effectiveness of silver nanoparticles (AgNPs)^16,30,31^, silver nanoparticles aggregation reduces the surface area of the nanoparticles and the emission of silver ions, reducing the antimicrobial effect of the particles^27,31–33^. Holtz *et al*.^24^ found that, in addition to a possible antibacterial action, silver vanadate acts as a carrier for the silver nanoparticles, allowing a continuous release of silver ions, and avoiding possible accumulations of AgNPs, and consequently maintaining a large surface area.

In this study, the resin liner incorporated with 1 and 2.5% AgVO_3_ showed antimicrobial activity for *E*. *faecalis*, and 5 and 10% for *E*. *faecalis*, *P*. *aeruginosa*, and *C*. *albicans*. None of the concentrations was effective against *S*. *aureus* (Fig. 4).

The cell wall of Gram-negative bacteria comprises a fine peptidoglycan sacculus which is covered by an outer membrane. In contrast, Gram-positive bacteria have the much thicker cell wall, with multiple layers of peptidoglycan, and the presence of additional glycolipers in addition to other protective surface structures^34,35^. These additional glycopolymers are essential in maintaining the bacterial architecture, replication and other major cell functions, are highly diversified and generally species or strain-specific. Most Gram-positive bacteria have two types of glypolymers, one peptidoglycan-attached and one lipid-attached, although some bacterial species have three or four different glypolymers^35^.

Our research evidenced the presence of a different antibacterial effect for two of the gram positive bacteria tested, with effect for *E*. *faecalis* and no effect for *S*. *aureus*. This difference may be related to the presence of different additional glypolymers in the cell wall of both bacteria. The effect of AgVO_3_ on P. *aeruginosa* is probably due to the thin cell wall, characteristic of gram negative bacteria.

According to Holtz *et al*.^23^ the contact of AgNPs change the bacterial membrane morphology, and when bound to DNA, they affect bacterial metabolic processes, in particular cell division, causing cell death. As for *C*. *albicans*, AgNPs function by breaking down the membrane potential and forming pores, causing leakage of ions and other materials inducing apoptosis and causing ultrastructural changes^36,37^.

In one of our previous studies^22^ where cell metabolism analysis was performed using the XTT method, a decline in the metabolism of *C*. *albicans* was observed with the incorporation of 5 and 10% b-AgVO_3_ and in the present study, 5 and 10% promoted antimicrobial effect.

Several studies^1,16,20,27,28^ using antimicrobial agents such as silver zeolite^1^, silver nanoparticles^16^, Plant-Derived Component^6,20,27,28^, drugs^38^, and photocatalysts^14^ found activity against *C*. *albicans*, gram-positive, and gram-negative bacteria. However, some disadvantages were encountered such as short duration of antimicrobial capacity^14^, and alterations in the material’s roughness^14^, hardness^18^, fluidity^28^, adhesion^6,19,39^ and gellyfication properties^6^.

Comparisons of the mechanical properties of modified denture liners among studies are difficult because of the various methods used to evaluate the properties^7,18,39,40^. Trusoft has been tested for different properties such as porosity^7^, hardness^18,39^, absorption^39^, solubility^39^, and traction^39^. Although the composition of Trusoft is not specified by the manufacturer, its liquid component contains ethyl alcohol and low concentration of plasticizers, which guarantees a clinically acceptable smoothness for up to 6 months.

Soft denture liners should exhibit low hardness levels^18,39^, however, during use, they are susceptible to changes related to the leaching of plasticizers^40^. Mancuso *et al*.^39^ found that thermocycling exerted a significant effect on the hardness of Trusoft due to the hydrophilic character of the material, which can lead to hardening through ethanol loss, water absorption, and loss of the plasticizer^7,41^. Urban *et al*.^18^ observed a hardness increase of Trusoft when antimicrobial drugs were added, but the change was insufficient to interfere with clinical use. In the present study, the incorporation of 1% AgVO_3_ produced specimens with hardness values in compliance with ISO requirements for soft liners (ISO 10139-2: 2009)^40^ of approximately 40 units (DIN 53505 and ASTM D2240/75).

The bond strength of AgVO_3_-modified Trusoft to the heat-curing resin was higher for the 2.5 and 10% concentration, compared to the control group. In the attempt to elucidate this difference in the tensile strengh values, the tensile test was repeated for the 5% group, however, the same results were found. We suggest in a future study the accomplishment of the microstructural characterization proposing to analyze the structure-properties correlation in the attempt to find the answer for this difference in tensile strength means values for groups 2.5 and 10%.

Similar results were reported with the addition of 40 ppm silver nanoparticles (1.33 ± 0.22 MPa) compared to control (1.18 ± 0.17 MPa)^19^. In contrast, different results have also been found, where the control group (0.91 ± 0.52 N) presented greater tensile strength compared to the modified group (0.16 ± 0.05 N)^6^.

A previous study has reported a decrease of adhesive strength and changes in the type of failure with the increase of silver nanoparticles concentration^13^. In this study, the heat-curing resin had 50% of mixed failures in the 2.5% AgVO_3_ group and 60% of adhesive failures in the 10% group. Takahashi *et al*.^38^ found cohesive and mixed failures and a lower tensile strength for Trusoft without additives, compared to our results. The authors believed the findings were related to the high elasticity of the liner rather than bonding deficiencies. They also associated the failure of the liner to the low cohesive resistance of the material, which in turn provides information on the strength of the liner, rather than an accurate measure of the bond strength between liner and denture material^38^.

In the present study, the incorporation of different concentrations of AgVO_3_ did not affect roughness, which is an important parameter, since the incorporation of antimicrobials to denture liners may increase the recommended roughness parameter of 0.2 μm^42,43^.

The properties evaluated in this study were selected due to their clinical influence. An appropriate hardness allows the material to provide cushioning for the supporting mucosa. Adequate adhesion to the prosthesis and low levels of roughness are important to avoid the adhesion and proliferation of microorganisms.

## Conclusions

The incorporation of 5% AgVO_3_ to a soft denture liner was efficient in the control of *P*. *aeruginosa*, *E*. *faecalis*, and *C*. *albicans*, and 2.5 and 10% improved the adhesion properties between the liner and the denture base material. There was no effect on roughness and the 1% concentration maintained the recommended hardness properties of a soft material (ISO 10139-2: 2009). In conclusion, the AgVO_3_-incorporated denture liner is an efficient, practical, effective and accessible alternative for denture users with an oral infection.
